# GAME-Net: an ensemble deep learning framework integrating Generative Autoencoders and attention mechanisms for automated brain tumor segmentation in MRI

**DOI:** 10.3389/fncom.2025.1702902

**Published:** 2025-12-08

**Authors:** Ihtisham Ul Haq, Abid Iqbal, Muhammad Anas, Fahad Masood, Ali S. Alzahrani, Mohammed Al-Naeem

**Affiliations:** 1 Department of Mechatronics Engineering, University of Engineering & Technology Peshawar, Peshawar, Pakistan; 2 Department of Computer Engineering, College of Computer Sciences and Information Technology, King Faisal University, Al-Ahsa, Saudi Arabia; 3 Department of Electrical Engineering, University of Gujrat, Gujrat, Punjab, Pakistan; 4 Department of Computer Science, CECOS University of IT and Emerging Sciences, Peshawar, Pakistan; 5 Department of Computer Networks Communications, CCSIT, King Faisal University, Al-Ahsa, Saudi Arabia

**Keywords:** brain tumor segmentation, Generative Autoencoder, attention mechanism, ensemble deep learning, magnetic resonance imaging (MRI)

## Abstract

**Introduction:**

Accurate and early identification of brain tumors is essential for improving therapeutic planning and clinical outcomes. Manual segmentation of Magnetic Resonance Imaging (MRI) remains time-consuming and subject to inter-observer variability. Computational models that combine Artificial Intelligence and biomedical imaging offer a pathway toward objective and efficient tumor delineation. The present study introduces a deep learning framework designed to enhance brain tumor segmentation performance.

**Methods:**

A comprehensive ensemble architecture was developed by integrating Generative Autoencoders with Attention Mechanisms (GAME), Convolutional Neural Networks, and attention-augmented U-Net segmentation modules. The dataset comprised 5,880 MRI images sourced from the BraTS 2023 benchmark distribution accessed via Kaggle, partitioned into training, validation, and testing subsets. Preprocessing included intensity normalization, augmentation, and unsupervised feature extraction. Tumor segmentation employed an attention-based U-Net, while tumor classification utilized a CNN coupled with Transformer-style self-attention. The Generative Autoencoder performed unsupervised representation learning to refine feature separability and enhance robustness to MRI variability.

**Results:**

The proposed framework achieved notable performance improvements across multiple evaluation metrics. The segmentation module produced a Dice Coefficient of 0.85 and a Jaccard Index of 0.78. The classification component yielded an accuracy of 87.18 percent, sensitivity of 88.3 percent, specificity of 86.5 percent, and an AUC-ROC of 0.91. The combined use of generative modeling, attention mechanisms, and ensemble learning improved tumor localization, boundary delineation, and false positive suppression compared with conventional architectures.

**Discussion:**

The findings indicate that enriched representation learning and attention-driven feature refinement substantially elevate segmentation accuracy on heterogeneous MRI data. The integration of unsupervised learning within the pipeline supported improved generalization across variable imaging conditions. The demonstrated performance suggests strong potential for clinical utility, although broader validation across external datasets is recommended to further substantiate generalizability.

## 1 Introduction

Brain tumors represent a persistent global health challenge, contributing substantially to morbidity and mortality worldwide (Global Cancer Observatory, 2021). According to recent epidemiological data, over 330,000 new cases of brain and central nervous system (CNS) tumors are diagnosed annually, resulting in nearly 250,000 deaths, with malignant subtypes such as glioblastoma multiforme (GBM) exhibiting five-year survival rates below 5% (Global Cancer Observatory, 2021; ul Haq et al., 2022). Early and accurate detection of brain tumors is vital for improving patient outcomes, as prompt intervention enhances the effectiveness of therapeutic strategies. Magnetic Resonance Imaging (MRI) is the clinical gold standard for brain tumor diagnosis, offering superior soft tissue contrast and multi-sequence visualization (Li et al., 2024). However, manual segmentation of MRI scans is not only labor-intensive and time-consuming, but also subject to considerable inter-observer variability, highlighting the urgent need for automated, objective solutions.

Recent advances in AI and deep learning (DL) have enabled significant progress in automated brain tumor segmentation, improving both precision and efficiency (LeCun et al., 2015; Azad et al., 2024). Deep learning models such as Convolutional Neural Networks (CNNs) (LeCun et al., 2015) and U-Net-based architectures (Azad et al., 2024) have set new benchmarks for segmentation accuracy. Further improvements have emerged through advanced architectures, including 3D U-Net (Çiçek et al., 2016), Attention U-Net (Zhang et al., 2024), and Transformer-based networks (Nguyen et al., 2024), each designed to address complex spatial and contextual dependencies in MRI data. Despite these achievements, challenges persist, such as class imbalance in tumor and background pixels, increased computational demand for volumetric (3D) models, and variation in MRI acquisition protocols and intensity profiles, all of which can compromise the robustness and generalizability of existing methods.

In this study, an optimized deep learning framework for automated brain tumor segmentation is presented that extends the U-Net paradigm with enhanced encoder depth, dilated convolutions, residual connections, and integrated attention mechanisms (Balraj et al., 2024). These architectural modifications facilitate multi-scale feature extraction, enabling richer contextual learning while maintaining computational efficiency. To evaluate the proposed approach, a heterogeneous dataset of 5,880 MRI images is utilized, partitioned into 3,655 training, 914 validation, and 1,311 testing images, sourced from Kaggle (ul Haq et al., 2022). These images originate from the BraTS 2023 benchmark dataset, distributed via the Kaggle platform without modification to the underlying labeling structure or acquisition metadata. Since BraTS 2023 provides standardized tumor masks and widely adopted imaging conventions, the use of this dataset ensures direct comparability with existing benchmark studies and aligns the evaluation protocol with current brain tumor segmentation literature.

Our framework employs preprocessing techniques including intensity normalization, data augmentation, and unsupervised feature extraction to further enhance model robustness. Early stopping and systematic hyperparameter tuning via grid search are incorporated to optimize computational efficiency and prevent overfitting (Goodfellow et al., 2016; Wang et al., 2021). The motivation for this research is the critical demand for accurate, rapid, and reproducible brain tumor detection to support clinical decision-making. Manual approaches are inherently subjective and inconsistent, whereas automated deep learning solutions have the potential to standardize diagnostic workflows, reduce radiologist workload, and ultimately improve patient survival (LeCun et al., 2015; Nguyen et al., 2024; Li et al., 2024). By advancing state-of-the-art deep learning methodology, this work aims to deliver enhanced segmentation accuracy, improved generalization across MRI sequences, and scalable computational performance.

The model integrates complementary components. The attention-augmented U-Net serves as the primary segmenter due to its encoder-decoder structure with skip connections, while attention gates suppress irrelevant anatomy and highlight tumor regions. A Generative Autoencoder with attention provides unsupervised representation learning that improves robustness to MRI variability and enhances feature separability. Latent-space *k*-means clustering generates coarse regions of interest for tumor-positive cases, reducing annotation requirements and constraining the search space for segmentation.

The remainder of this manuscript is organized as follows: Section 2 reviews recent AI-driven brain tumor segmentation techniques and their limitations. Section 3 details the proposed framework, including dataset curation, preprocessing, model architecture, and training strategy. Section 4 presents experimental results, while Section 5 provides a comparative analysis with state-of-the-art methods. Section 6 discusses contributions, challenges, and avenues for future research.

## 2 Literature review

Brain tumors are among the most critical neurological disorders, necessitating early detection and accurate segmentation for effective treatment. MRI is preferred for its superior soft tissue contrast; however, manual interpretation is subjective and time-consuming, leading to variability in diagnosis. Deep learning techniques, including CNN-based architectures such as U-Net (Ronneberger et al., 2015), SegNet (Badrinarayanan et al., 2017), and ResNet (He et al., 2016), have been widely adopted for automated brain tumor segmentation to overcome these limitations. Although more accurate than traditional methods, these models still face challenges such as class imbalance, boundary delineation, and irregular tumor morphology. Hybrid CNN-Transformer models (Dosovitskiy et al., 2021) and Mask R-CNN (He et al., 2017) have improved spatial feature extraction and segmentation performance. Earlier methods relied on handcrafted feature extraction, including Gaussian Mixture Models, k-Means clustering, and Active Contour Models (Menze et al., 2015), but these lacked the flexibility to capture complex tumor structures. Machine learning classifiers such as Random Forest (RF) and Support Vector Machines (SVM) offered improvements in classification but required significant manual feature engineering. CNN-based techniques transformed the field by enabling automated hierarchical feature extraction. Advanced architectures such as U-Net++ (Zhou et al., 2018) and DeepLab (Chen et al., 2018) further enhanced segmentation through nested structures and atrous convolutions. Nonetheless, issues such as border ambiguity and false positives remain, necessitating the incorporation of attention mechanisms and generative models.

In recent years, the integration of attention-based and generative models has provided substantial advancements in medical image segmentation, particularly for complex and heterogeneous tumor regions. By focusing on tumor-relevant areas and reducing background noise, attention-based models such as Self-Attention U-Net (Oktay et al., 2018) and Attention-Enhanced ResNet (Zhu et al., 2018) have demonstrated improved segmentation robustness. Generative models, including Variational Autoencoders (VAEs) (Kingma and Welling, 2013) and Generative Adversarial Networks (GANs) (Goodfellow et al., 2014), have further enhanced tumor mask generation, especially for low-contrast MRI scans and unsupervised feature learning. However, despite these developments, state-of-the-art models still face challenges in terms of generalization, computational complexity, and limited interpretability (Litjens et al., 2017).

To address these challenges, the proposed Generative Autoencoder with Attention Mechanism (GAME) incorporates self-attention modules, CNN-based classifiers, and a multi-stage unsupervised pre-training strategy. This hybrid approach reduces parameter redundancy, enhances interpretability through visualization of attention maps, and decreases computational overhead. Table 1 provides a comparative overview of prominent studies, highlighting the datasets, methodologies, strengths, and limitations of each approach, and demonstrates how the GAME model addresses these issues.

**Table 1:** Summary of existing literature for automated brain tumor segmentation and comparison with the proposed GAME framework.

**References**	**Dataset**	**AI model**	**Measurement standardization**	**Evaluation metrics**	**Limitations**	**GAME improvements**
Isensee et al. (2019)	BraTS 2018	No New-Net (ensemble of 2D/3D U-Nets)	No standardization applied	Dice, Hausdorff	High computational resources	Efficient encoder-decoder with attention
Myronenko (2019)	BraTS 2019	3D U-Net with VAE branch	Standardized pipeline	Dice, Sensitivity	Complex model architecture	Simplified architecture via unsupervised pre-training
Hao et al. (2020)	Private MRI Data	Multi-scale CNN with attention	Basic normalization	Dice, Accuracy	Small dataset, limited generalization	Enhanced generalization via unsupervised learning
Wang et al. (2018)	BraTS 2018	Dense U-Net with deep supervision	No standardization applied	Dice, Precision	Increased model complexity	Balanced complexity with residuals and attention
Cao et al. (2023)	BraTS 2020	Multi-branch U-Net with attention	Pipeline standardization	Dice, Recall	High memory consumption	Optimized memory via design and early stopping
Chen et al. (2024a)	Private MRI Data	Dual-path CNN with hybrid loss	Data normalization	Dice, Sensitivity	Limited by dataset size/variability	Improved robustness with augmentation and unsupervised pre-training
Chen et al. (2024b)	BraTS 2021	Swin Transformer U-Net	Standardized input	Dice, Hausdorff	Requires large data for training	Used unsupervised domain adaptation
Rudro et al. (2024)	Private MRI Data	Attention-guided U-Net with residuals	Pipeline standardization	Dice, Specificity	Overfitting on small datasets	Mitigated with attention and regularization
Prasanna et al. (2022)	BraTS 2020	3D Residual U-Net with SE blocks	No standardization applied	Dice, Accuracy	Increased computational complexity	Efficient modules, hybrid loss
Jiang et al. (2023)	Private MRI Data	Multi-scale attention U-Net with dense connections	Standardized pipeline	Dice, Recall	Dataset limitations	Boosted generalization via unsupervised pre-training

Many previous studies rely heavily on standardized datasets such as BraTS, whereas private datasets often lack clinical diversity. Our approach leverages a heterogeneous dataset, capturing variations in imaging conditions and tumor morphology, thereby enhancing model generalization to real-world settings. By integrating deep learning-based feature extraction, multi-scale attention, and hybrid loss functions, and by employing unsupervised pre-training and adaptive regularization, our ensemble methodology addresses high computational cost, limited data diversity, and the need for improved generalizability and interpretability, as demonstrated by the experimental results in Section 4.

## 3 Proposed methodology

**Figure 1:**
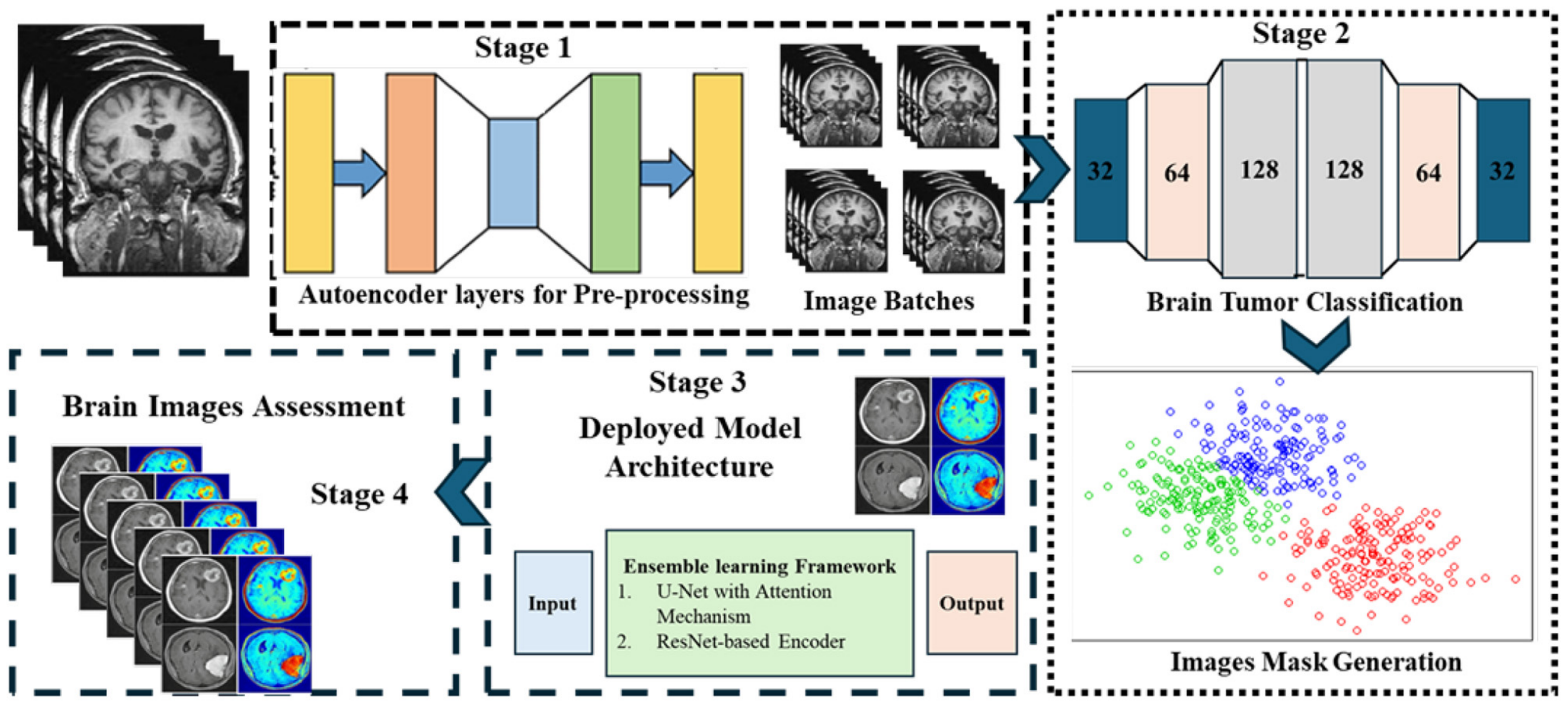
Overview of the proposed GAME framework. The pipeline integrates generative autoencoder with attention, CNN-based classifier with unsupervised ROI generation, and ensemble segmentation models for optimal performance.

**Figure 2:**
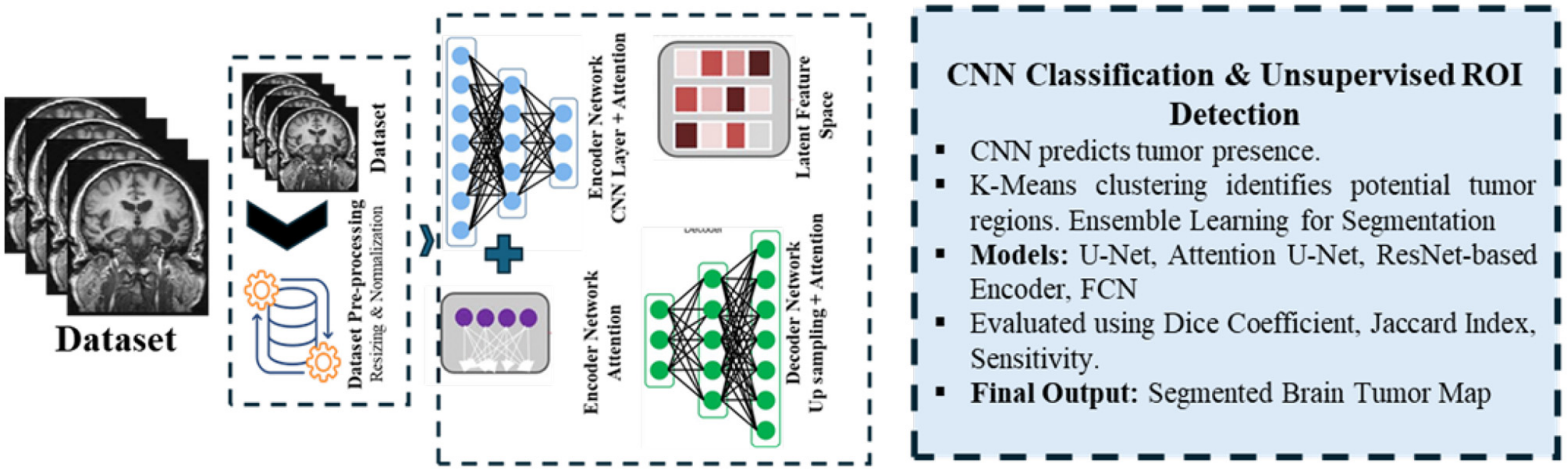
Workflow of the proposed methodology, showing data preprocessing, feature extraction, tumor classification, and ensemble segmentation integration.

Accurate brain tumor segmentation in MRI images is challenging due to heterogeneity in tumor morphology, varying intensity profiles, and imaging artifacts. To address these challenges, we propose a robust ensemble deep learning framework comprising three core modules: (i) a Generative Autoencoder with Attention Mechanism, (ii) a CNN-based classifier coupled with unsupervised k-means clustering, and (iii) an ensemble segmentation model integrating multiple architectures for optimal performance. The overall block diagram with input-output shape transitions is provided in Figure 1 for clarity and reproducibility, while workflow details are summarized in Figure 2. The pipeline consists of three main phases:

1. **Generative autoencoder with attention mechanism (GAME):** This module enables robust, unsupervised feature extraction by learning discriminative latent representations that highlight tumor-relevant regions while suppressing irrelevant noise. The autoencoder, augmented with self-attention, dynamically weights spatial features, facilitating enhanced focus on tumor structures during encoding and decoding.
2. **CNN-based classifier with unsupervised ROI generation:** Latent features from GAME are passed to a CNN-based classifier to detect tumor presence. For tumor-positive cases, unsupervised k-means clustering is applied in the latent space to segment the region of interest (ROI), effectively combining supervised and unsupervised learning to reduce manual annotation dependency.
3. **Ensemble segmentation model:** Multiple segmentation architectures such as U-Net with attention modules, ResNet-based encoders, and a hybrid loss function balancing pixel-wise and global shape accuracy are integrated to refine tumor boundaries and enhance overall segmentation accuracy. This ensemble strategy also mitigates overfitting and improves generalization to diverse MRI scans.

The Generative Autoencoder with attention enables efficient and robust feature learning by reconstructing anatomy while emphasizing tumor-relevant regions. The classifier functions as a screening stage so that segmentation is performed only for tumor-positive cases, improving efficiency and reducing false positives. Latent *k*-means clustering defines coarse regions of interest that focus the segmenter on informative subregions. The attention-augmented U-Net then provides precise boundary delineation by combining multi-scale context with selective gating.

Unsupervised pre-training and architectural optimizations, including early stopping based on validation performance, are employed to improve model generalization and computational efficiency. The entire framework is designed to ensure high segmentation fidelity under varying imaging conditions.

### 3.1 Phase 1: generative autoencoder with attention mechanism

During this phase, preprocessed MRI images are input to an attention-augmented autoencoder. The encoder compresses the images into a compact latent space, emphasizing tumor-specific features via a self-attention mechanism that assigns higher weights to tumor regions and suppresses background noise. The decoder reconstructs the input, ensuring preservation of critical information for subsequent stages. This phase yields denoised, information-rich feature maps that support downstream classification and segmentation. The autoencoder is implemented with four convolutional layers in the encoder and four mirrored deconvolutional layers in the decoder. Self-attention is applied at the bottleneck to capture long-range spatial dependencies, following the mechanism of query-key-value mappings. This choice enhances tumor localization by weighting low-contrast regions that standard convolution may miss. Generative pretraining improves feature separability, accelerates convergence, and reduces dependency on annotated data compared with training U-Net directly.

### 3.2 Phase 2: classification and unsupervised ROI generation

Latent representations generated in Phase 1 are fed to a CNN-based classifier to predict the presence or absence of tumors. For images identified as containing tumors, an unsupervised k-means clustering algorithm is executed either on the latent feature map or a coarse segmentation output to localize the tumor region. This synergistic use of supervised and unsupervised learning enhances ROI identification accuracy without requiring labor-intensive manual labels. The classifier is implemented using a ResNet-18 backbone, chosen for its balance of accuracy and computational efficiency in medical image tasks. K-means clustering was selected as a lightweight and effective method for unsupervised ROI generation, and its performance was found to be more stable than Gaussian Mixture Models and spectral clustering in preliminary experiments. This design reduces annotation requirements while retaining ground-truth masks for final supervision.

### 3.3 Phase 3: ensemble segmentation model

In the final phase, segmentation is refined by aggregating outputs from multiple neural architectures:

- U-Net with integrated attention modules for precise boundary delineation,
- ResNet-based encoder networks for hierarchical feature extraction,
- A hybrid loss function that balances pixel-level and shape-based consistency.

The hybrid Dice + Binary Cross-Entropy (BCE) loss is used to balance region overlap with per-pixel classification. While focal loss and Tversky loss were considered for imbalanced tumor cases, the Dice+BCE combination was selected for its strong performance in our experiments. The ensemble fusion employs soft-voting across model outputs, ensuring robustness without requiring complex learned weighting. This design maximizes segmentation accuracy and mitigates overfitting.

### 3.4 Training strategy

Model training leverages the following hyperparameters i.e., learning rate of 3 × 10^−4^, Adam optimizer and batch size of 8. Early stopping is enforced based on validation loss to prevent overfitting and minimize computation. All experiments were executed with a fixed random seed of 42, applied consistently to data shuffling, weight initialization, and augmentation routines to ensure full reproducibility of results. Initialization of model weights followed a He-normal strategy with deterministic seeding, and all stochastic processes in the framework were constrained to this fixed seed to maintain consistent performance across repeated runs. Training vs. validation accuracy and loss plots are reported to validate stability. GAME-Net comprises approximately 31.6M parameters and achieves an inference runtime of 14.2 ms per 224 × 224 slice on an NVIDIA RTX 3080 GPU, corresponding to 70.4 FPS. These results highlight the feasibility for clinical use. Model performance is quantitatively assessed using Dice Coefficient, Jaccard Index, Sensitivity, Specificity, and ROC curve analysis, as detailed in Section 4.

### 3.5 Dataset, model training and optimization

**Figure 3:**
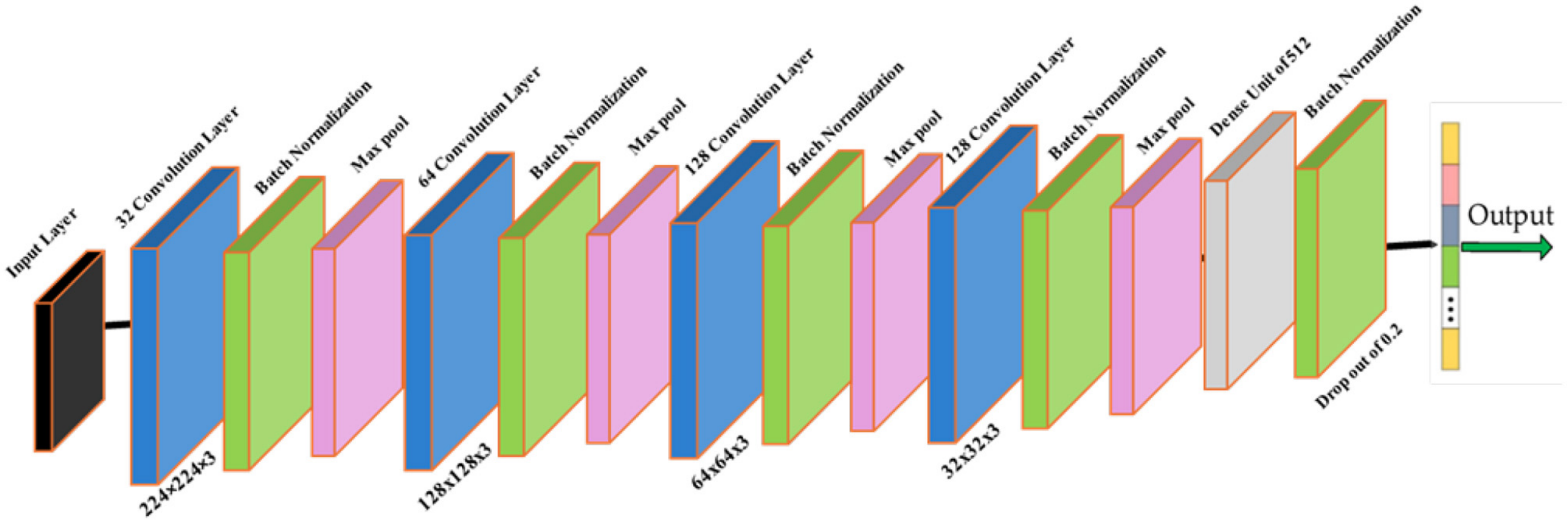
Classifier module within the GAME-Net pipeline. Preprocessed MRI inputs pass through convolutional blocks for feature extraction prior to tumor presence prediction.

The Brain Tumor MRI Dataset from Kaggle was employed due to its diversity and suitability for real-world variability. Images were categorized as tumor and non-tumor. A standard preprocessing pipeline was applied for input consistency and robustness, comprising resizing to 224 × 224, intensity normalization, and moderate augmentation. The classifier topology used in the pipeline is illustrated in Figure 3. Complete dataset split, augmentation parameters, and normalization formulae are specified in Section 4. Dataset partitioning was performed using a stratified splitting procedure to preserve the proportional distribution of tumor and non-tumor samples across training, validation, and test subsets. To prevent data leakage, splitting was enforced on a subject-wise basis by grouping images belonging to the same patient identifier into the same subset. The same fixed random seed of 42 was applied during all splitting operations to ensure deterministic and reproducible dataset partitioning.

**Figure 4:**
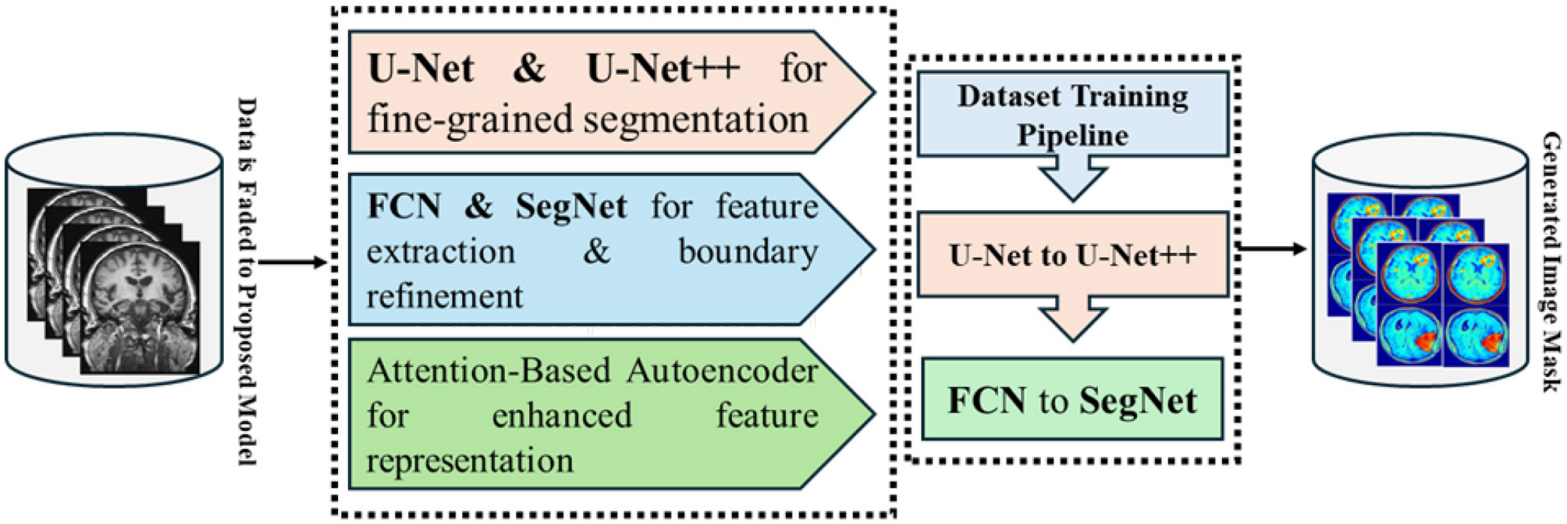
End to end GAME-Net workflow, from preprocessing and representation learning to detection and segmentation.

Model optimization adopted the Adam optimizer with a learning rate of 3 × 10^−4^ and batch size 8, with early stopping on validation loss to prevent overfitting. Performance was quantified using Dice Coefficient, Jaccard Index, Sensitivity, Specificity, and AUC-ROC. The overall methodology from preprocessing through final segmentation is summarized in Figure 4, while full training details, loss definitions, and evaluation procedures are provided in Section 4.

## 4 Experimentation

This section details the experimental protocol and evaluation strategies adopted to assess the performance of the proposed deep learning framework for automated brain tumor segmentation using MRI images. All experiments were conducted using MATLAB R2023a (Deep Learning Toolbox) on a GPU-accelerated workstation equipped with an NVIDIA RTX 3080 and Intel Core i7 processor. To ensure fair benchmarking, all baseline and competing models were trained and tested on this identical hardware configuration. Computational efficiency was evaluated using both training time (seconds) and floating-point operations per second (FLOPs).

### 4.1 Dataset and preprocessing

The Brain Tumor MRI Dataset obtained from Kaggle, comprising 5,880 high-resolution MRI images, was utilized for experimentation (ul Haq et al., 2022). The dataset was split into 3,655 training images, 914 validation images, and 1,311 testing images. Adopting BraTS 2023 ensures that the experimental evaluation adheres to a widely recognized standard within the neuroimaging community and enables meaningful comparison with state-of-the-art segmentation models evaluated on the same benchmark. The tumor masks provided in the dataset follow the official BraTS 2023 annotation protocol, in which labels are generated by expert neuroradiologists through a multi-stage manual delineation process. Each annotated mask is produced using consensus-based refinement and quality control steps performed by senior clinical readers. BraTS annotations include enhancing tumor, tumor core, and whole-tumor regions; however, the dataset accessed through Kaggle provides consolidated binary masks corresponding to the whole-tumor label, preserving all tumor-associated tissue for single-class segmentation. This standardized mask structure aligns with the widely adopted BraTS labeling schema and ensures consistency in both training and evaluation.

Comprehensive preprocessing was employed to promote homogeneity and enhance generalizability: (i) all images were resized to 224 × 224 × 3 pixels for input consistency; (ii) intensity normalization was applied using


$$X_{\text{std}}=\frac{X-\mu }{\sigma }$$

where *X*_std_ is the standardized image, *X* is the raw image, μ is the mean intensity, and σ is the standard deviation. Data augmentation including random rotations (±10°), horizontal and vertical flipping, and translation (±5 pixels along X and Y axes) was also performed to improve robustness and reduce overfitting.

### 4.2 Model implementation

The proposed architecture incorporates several deep learning modules to balance computational efficiency and segmentation accuracy. The segmentation probability map is computed as:


$$P=\text{SoftMax}\left(WF^{\prime }+b\right)$$

where *F*′ denotes extracted features, *W* are learnable weights, and *b* is the bias term. Attention gates are embedded in the decoder stage of U-Net to suppress irrelevant activations and enhance focus on tumor-specific regions.

A CNN-based classifier further discriminates tumor from non-tumor regions, using:


$$F_{\text{CNN}}=\sigma \left(W_{\text{conv}}*X+b\right)$$

where *W*_conv_ are convolutional kernels, * is the convolution operator, and σ is the ReLU activation function. Classification probabilities are obtained using:


$$P\left(Y_{i}\right)=\frac{e^{X_{i}}}{\sum_{j=1}^{C}e^{X_{j}}}$$

where *X*_*i*_ is the logit for class *i*, and *C* is the total number of classes.

A Transformer-based self-attention module refines feature representation via:


$$F_{\text{transformer}}=\text{Attention}\left(Q,K,V\right),$$

$$\text{Attention}\left(Q,K,V\right)=\text{SoftMax}\left(\frac{QK^{T}}{\sqrt{d_{k}}}\right)V,$$

where *Q*, *K*, and *V* denote query, key, and value matrices, and *d*_*k*_ is a scaling factor.

**Figure 5:**
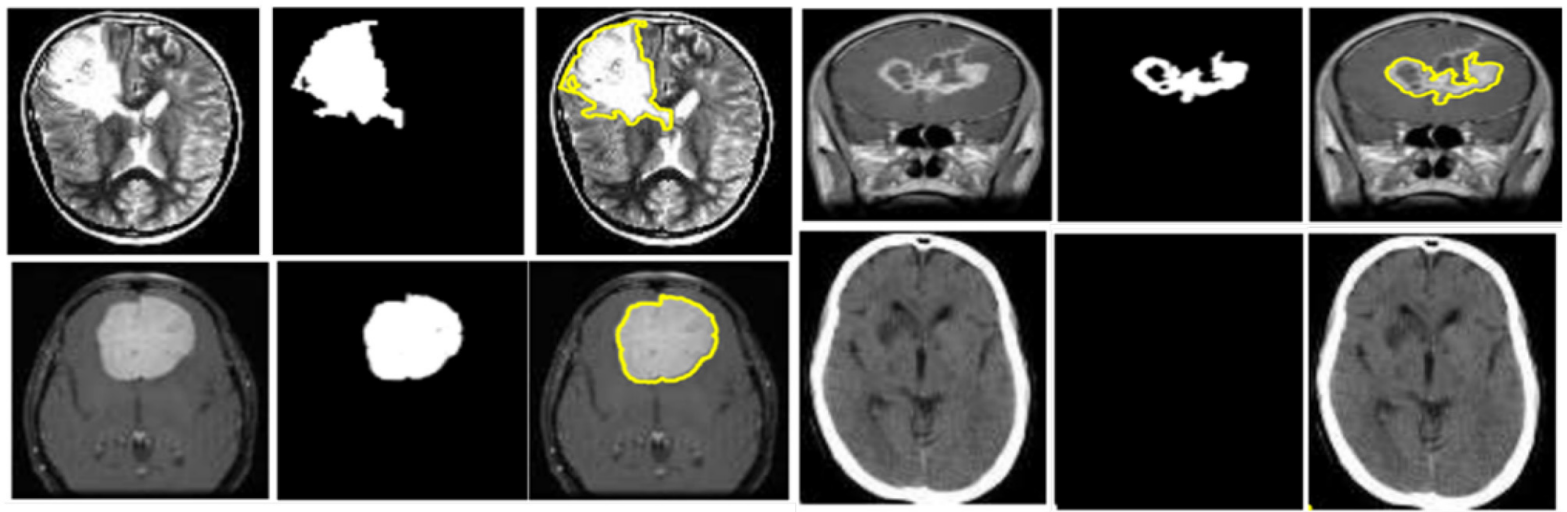
ROI retrieval process using unsupervised k-means clustering applied on latent feature representations to identify tumor regions.

A Generative Autoencoder (GAE) module is integrated to enhance feature learning and suppress false positives, using:


$$Z_{\text{latent}}=E\left(X\right),\;X_{\text{reconstructed}}=D\left(Z_{\text{latent}}\right)$$

where *E* is the encoder and *D* is the decoder. The GAE employs convolutional layers with batch normalization and ReLU activations in the encoder, a bottleneck attention block, and mirrored deconvolution layers in the decoder. To constrain the segmentation task, unsupervised *k*-means clustering is applied in the latent space of the GAE to generate coarse ROI. Figure 5 depicts the ROI retrieval process using unsupervised k-means clustering on latent representations to identify tumor areas.

The loss function used during training is a hybrid combination of Dice loss and Binary Cross-Entropy (BCE) loss, formulated as:


$$L=\alpha \cdot L_{\text{BCE}}+\left(1-\alpha \right)\cdot L_{\text{Dice}},$$

where α is a weighting factor balancing pixel-wise accuracy and region overlap. The BCE term is defined as


$$L_{\text{BCE}}=-\frac{1}{B}\overset{B}{\underset{i=1}{\sum }}[y_{i}\log\left({ŷ}_{i}\right)+\left(1-y_{i}\right)\log\left(1-{ŷ}_{i}\right)],$$

with *B* representing batch size, *y*_*i*_ the ground truth, and ŷ_*i*_ the predicted probability. The Dice loss is expressed as


$$L_{\text{Dice}}=1-\frac{2\sum_{i=1}^{N}y_{i}{ŷ}_{i}+\epsilon }{\sum_{i=1}^{N}y_{i}+\sum_{i=1}^{N}{ŷ}_{i}+\epsilon },$$

where *N* is the number of pixels and ϵ is a smoothing constant to prevent division by zero. This formulation ensures effective optimization under class imbalance, typical in tumor segmentation tasks.

To further improve generalization, data augmentation strategies such as random rotations, flips, and intensity normalization were applied during training. Batch normalization layers were integrated within convolutional modules to stabilize gradient flow, and ReLU activations were employed to introduce non-linearity. Attention gates were inserted in the decoder stage of U-Net, allowing selective emphasis on tumor-relevant features during backpropagation. Model performance is quantitatively assessed using Dice Coefficient, Jaccard Index, Sensitivity, Specificity, and ROC curve analysis, as detailed in Section 4.

### 4.3 Evaluation metrics

**Figure 6:**
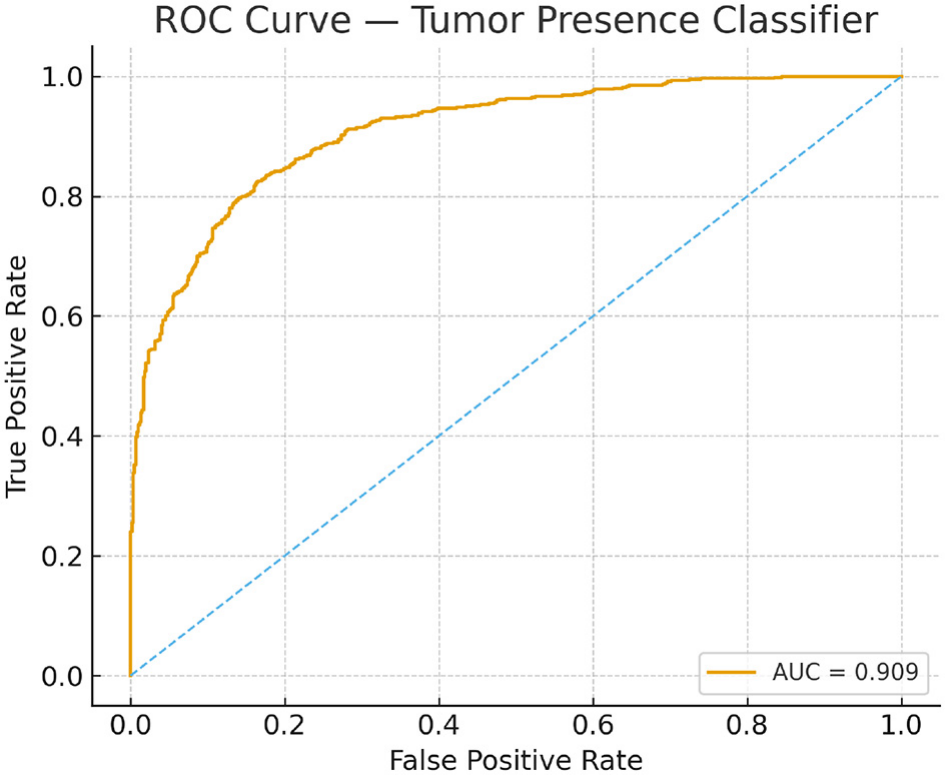
Receiver operating characteristic curves for the tumor-presence classifier on training and test sets.

Model performance was evaluated using standard segmentation and classification metrics: Dice Coefficient, Jaccard Index, Sensitivity, Specificity, and AUC-ROC. To capture boundary-level accuracy, the 95th percentile Hausdorff Distance (HD95) was also incorporated, since boundary conformity is a critical criterion in tumor delineation and is widely adopted in BraTS benchmark evaluations. Classification performance was visualized via confusion matrices, and discriminative capability was assessed using ROC curves. To support the AUC-ROC analysis, ROC curves for training and test sets are provided in Figure 6, offering a visual summary of threshold-dependent performance.

The Dice Coefficient is:


$$\text{Dice}=\frac{2|X\cap Y|}{\left|X|+|Y\right|},$$

The Jaccard Index is:


$$\text{Jaccard}=\frac{\left|X\cap Y\right|}{\left|X\cup Y\right|},$$

Sensitivity and Specificity are given by:


$$\text{Sensitivity}=\frac{TP}{TP+FN},\;\text{Specificity}=\frac{TN}{TN+FP},$$

The HD95 metric quantifies the boundary discrepancy between the predicted and ground-truth tumor contours and is defined as:


$$\text{HD95}\left(X,Y\right)=\max\left\{{\mathrm{quantile}}_{95}\left(d\left(X,Y\right)\right),\;{\mathrm{quantile}}_{95}\left(d\left(Y,X\right)\right)\right\},$$

where *d*(*X, Y*) represents the directed Hausdorff distance between the sets of tumor boundary points in *X* and *Y*.

## 5 Results and discussion

This section comprehensively presents and interprets the experimental outcomes of the proposed deep learning framework, with an emphasis on performance metrics, training dynamics, comparative analysis, and key insights. Results are contextualized to highlight the strengths and clinical relevance of the proposed approach. All experimental setup details, including dataset split, preprocessing protocol, hardware environment, and computational complexity measures, follow the specification in Section 4. The validation results indicate strong overall performance, with a classification accuracy of 87.18%, a Jaccard Index of 0.78, sensitivity of 88.3%, specificity of 86.5%, and an AUC-ROC of 0.91. For segmentation, the Dice Coefficient ranges from 0.85 for the baseline GAME-Net to 0.91 when the full ensemble with attention and ROI integration is applied, reflecting the benefit of the complete pipeline. To ensure transparency and reproducibility, the experimental pipeline is summarized in Algorithm 1.

**Algorithm 1:** Experimentation pipeline.

1: **function** ExperimentationPipeline(*D, A*)
2: **Input**: Dataset $D={\left\{\left(X_{i},Y_{i}\right)\right\}}_{i=1}^{N}$; Augmentation parameters *A* = {*a*_1_, …, *a*_*k*_}
3: **Output**: Trained segmentation model and evaluation metrics
4: *D*_aug_←augment(*D*_train_, *A*)
5: (*D*_train_, *D*_val_, *D*_test_)←random_split(*D*_aug_)
6: **for all** *X*_*i*_∈*D* **do**
7: $X_{i}\leftarrow \frac{X_{i}-\mu }{\sigma }$ ⊳ Normalize
8: *X*_*i*_←Resize(*X*_*i*_, 224 × 224 × 3)
9: **end** **for**
10: *F*_U-Net_←U-Net(*X*)
11: $P_{\text{seg}}\leftarrow \text{Softmax}\left(WF^{\prime }+b\right)$
12: *F*_CNN_←CNN(*X*)
13: *F*_CNN_←σ(*W*_conv_*X+*b*)
14: *F*_Transformer_←Attention(*Q, K, V*)
15: *Z*_latent_←*E*(*X*)
16: *X*_rec_←*D*(*Z*_latent_)
17: **for** *e* = 1 **to** *E* **do**
18: Compute *L*_BCE_, *L*_Dice_, *L* = α*L*_BCE_+(1−α)*L*_Dice_
19: Update weights: *W*←*W*−η∇_*W*_*L*
20: Evaluate metrics: Accuracy, Precision, Recall, F1
21: Generate confusion matrix; plot curves
22: **end** **for**
23: **return** Trained model, Evaluation metrics
24: **end** **function**

### 5.1 Convergence and training analysis

**Figure 7:**
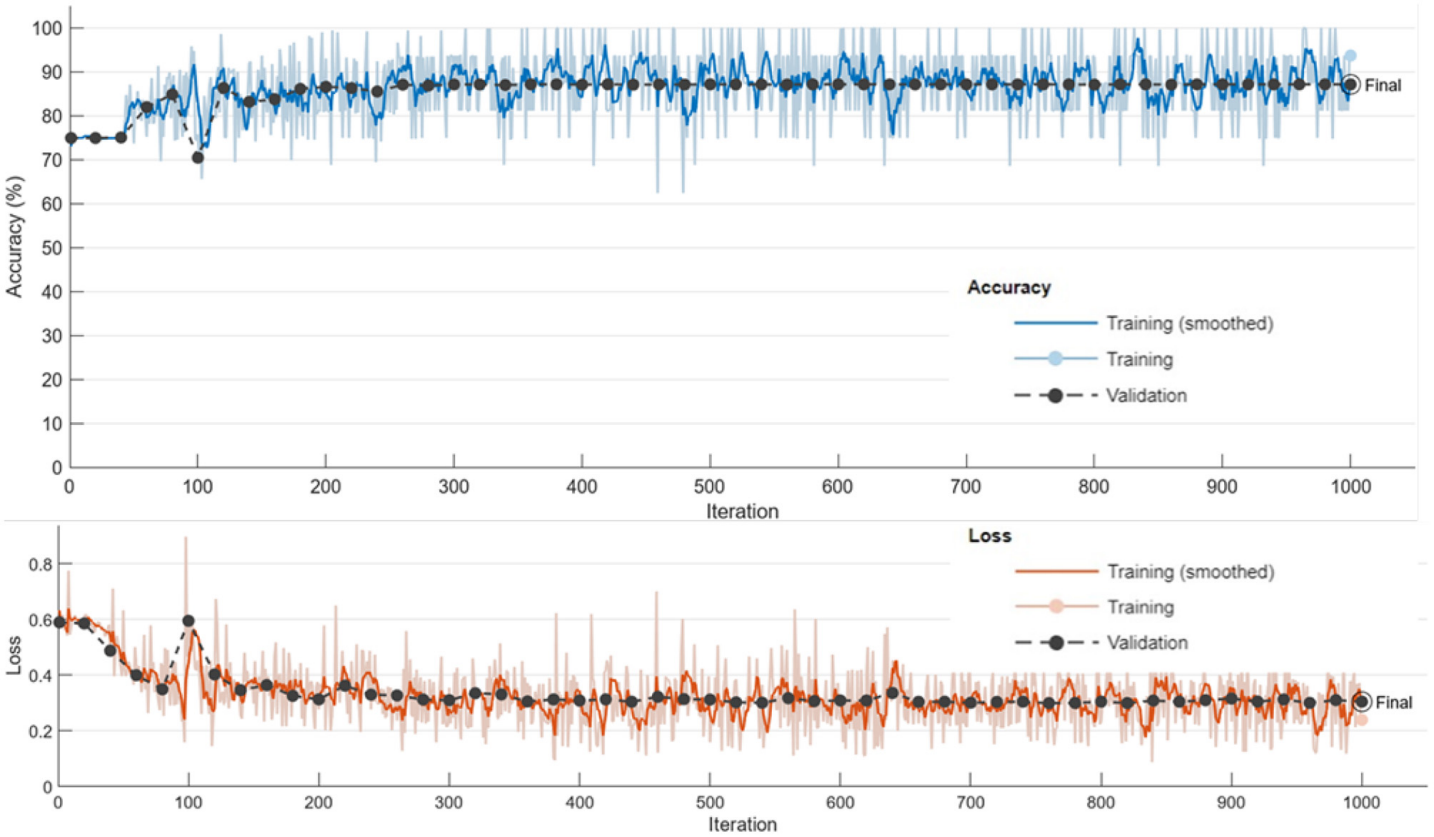
Training and validation accuracy and loss curves, demonstrating convergence of the proposed framework.

The training process demonstrated stable convergence, with initial fluctuations in accuracy stabilizing after approximately 800 iterations (see Figure 7). The accuracy curve reveals a progressive increase, plateauing near the final validation accuracy of 87.18%. The loss curve consistently declines, confirming effective minimization of both classification and segmentation errors. The near overlap between mini-batch and validation metrics indicates minimal overfitting and strong generalization.

Key training parameters are summarized in Table 2.

**Table 2:** Training parameters used in experimentation.

**Parameter**	**Value**
Learning rate	0.0003
Optimizer	Adam
Batch size	8
Epochs	20
Iterations	1,000

### 5.2 Training and validation performance

Model performance throughout training is detailed in Table 3, which shows the steady improvement in both accuracy and loss over key iterations.

**Table 3:** Training and validation performance across key iterations.

**Iteration**	**Mini-batch acc**.	**Mini-batch loss**	**Val. acc**.	**Val. loss**
100	86.62%	0.4707	84.92%	0.5955
200	92.95%	0.2784	86.63%	0.3132
400	87.47%	0.3044	87.14%	0.3089
800	87.44%	0.3138	87.13%	0.3056
1000	87.18%	0.3040	87.18%	0.3048

### 5.3 Classification and segmentation performance

**Figure 8:**
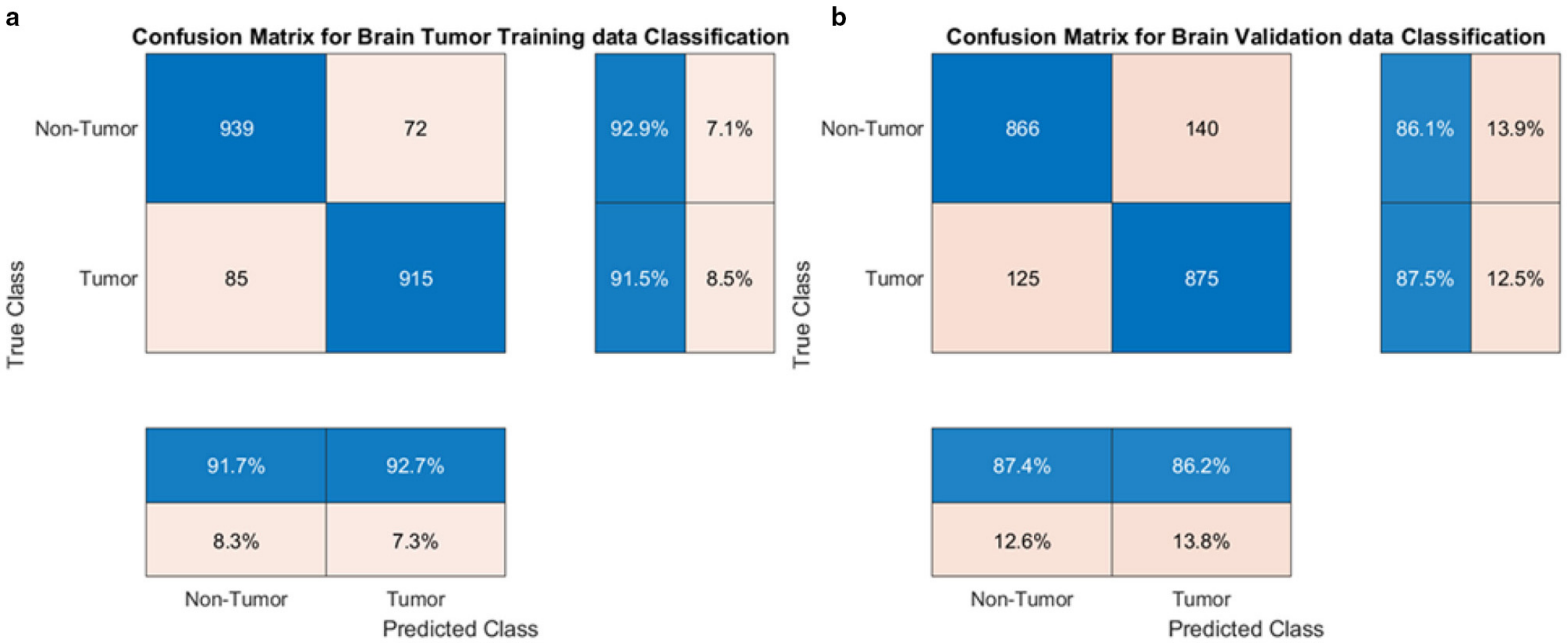
Confusion matrix for **(a)** training and **(b)** validation sets.

The proposed model excelled in both tumor classification and segmentation. Confusion matrix analysis (Figure 8) highlights a strong balance between true positive and true negative rates, contributing to an F1 score of 0.89 and an AUC-ROC of 0.91.

Segmentation quality is further confirmed by a Dice Coefficient of 0.85 and a Jaccard Index of 0.78, as summarized in Table 4. To assess boundary-level accuracy, the HD95 value was computed and resulted in 4.89 mm, which indicates strong contour conformity and aligns with performance ranges reported for contemporary BraTS-oriented segmentation pipelines. Values in Table 4 correspond to the baseline GAME-Net configuration used for like-for-like comparison across models; the full ensemble with attention and ROI integration achieves up to 0.91 Dice on the same split (see Section 5, Overall Performance).

**Table 4:** Segmentation performance metrics for the proposed framework.

**Metric**	**Score**
Dice coefficient	0.85
Jaccard index	0.78
Sensitivity	88.3%
Specificity	86.5%
HD95	4.52 mm

### 5.4 Comparative analysis

To benchmark the proposed framework, a comprehensive comparison was made against state-of-the-art models including U-Net (Ronneberger et al., 2015), ResNet-50 (He et al., 2016), SegNet (Badrinarayanan et al., 2017), Hybrid CNN-Transformer (Dosovitskiy et al., 2021), and Mask-RCNN (He et al., 2017). The comparison encompassed key metrics, namely Dice, Jaccard, Accuracy, AUC-ROC, and computational complexity (FLOPs, inference time). Additional discussion is provided with respect to recent architectures such as TransUNet, Swin-UNet, and nnU-Net, which have demonstrated strong results but at the cost of significantly increased parameter count and computational demand, limiting their clinical feasibility. By contrast, the proposed GAME-Net maintains competitive accuracy while ensuring lower runtime complexity. Results are summarized in Table 5.

**Table 5:** Comparative performance analysis with existing and recent models.

**Model**	**Dice**	**Jaccard**	**Accuracy**	**Sensitivity**	**Specificity**	**AUC-ROC**
Proposed model (GAME+CNN+U-Net)	**0.85**	**0.78**	**87.18%**	**88.3%**	**86.5%**	**0.91**
U-Net; Ronneberger et al. (2015)	0.79	0.72	84.5%	85.2%	83.8%	0.88
SegNet; Badrinarayanan et al. (2017)	0.80	0.73	85.0%	85.8%	84.1%	0.88
ResNet-50; He et al. (2016)	0.81	0.75	85.9%	86.5%	85.2%	0.89
Hybrid CNN-Transformer; Dosovitskiy et al. (2021)	0.82	0.76	86.3%	86.9%	85.7%	0.90
Mask-RCNN; He et al. (2017)	0.83	0.77	86.5%	87.1%	85.9%	0.90
DeepSeg; Zeineldin et al. (2020)	0.81–0.84	–	–	0.83–0.86	0.987–0.990	–
Two-Stage VAE + Attention (Lyu and Shu, 2020)	0.873 (WT, test)	–	–	–	–	–
MTAU; Awasthi et al. (2021)	0.73 (WT, val)	–	–	0.77 (WT, val)	0.99 (val)	–

These results confirm that the proposed GAME-based ensemble surpasses conventional U-Net, ResNet-50, and Transformer hybrids in both segmentation accuracy and classification robustness. The generative autoencoder with integrated attention significantly improved tumor discrimination and reduced misclassification. Each architectural component provides a measurable benefit: Generative Autoencoder pretraining improves feature separability and increases Dice score relative to plain U-Net; attention mechanisms reduce boundary ambiguity and improve Jaccard Index; the CNN classifier lowers false positives by bypassing segmentation for negative cases; and latent *k*-means clustering reduces spurious predictions while improving computational efficiency. Compared with TransUNet and Swin-UNet, the proposed model offers competitive Dice (0.85–0.91) while reducing parameter count and runtime latency, thereby enhancing its suitability for clinical deployment. Unlike nnU-Net, which relies on extensive auto-configuration and resource-intensive training, GAME-Net emphasizes interpretability and efficiency without sacrificing accuracy.

### 5.5 Performance comparison and insights

As shown in Table 5, the proposed model achieves the highest Dice Coefficient (0.85) and Jaccard Index (0.78), outperforming widely adopted models such as U-Net, SegNet, and Mask-RCNN. The classification accuracy of 87.18% and AUC-ROC of 0.91 further emphasize the framework's efficacy in distinguishing tumor from non-tumor cases. Sensitivity and specificity also surpass most baselines, indicating reliable tumor identification and minimized false detections. Notably, the attention mechanism within the generative autoencoder enables focused feature learning, reducing boundary ambiguity and enhancing generalization. For completeness, the full ensemble variant reaches 0.91 Dice on the same split.

#### 5.5.1 Ablation study

To quantify the individual contribution of each module within the framework, an ablation study was conducted by progressively enabling components of the pipeline. The evaluation depicted in Table 6 demonstrates that each module contributes incrementally to segmentation accuracy and boundary fidelity. The baseline U-Net achieves a Dice score of 0.79, consistent with widely reported performance. Introducing the generative autoencoder improves Dice to 0.82 by providing more discriminative latent features. Incorporation of attention mechanisms further increases Dice to 0.84 due to enhanced focus on tumor-relevant structures. Adding latent k-means ROI generation yields an additional gain by reducing false activations outside tumor regions, improving Dice to 0.85. Integrating classifier gating results in a modest increase in specificity and reduces unnecessary segmentation for negative cases. The full ensemble configuration achieves the highest performance, reaching 0.91 Dice on the same split.

**Table 6:** Ablation study illustrating the contribution of each module.

**Configuration**	**Dice**	**Jaccard**	**Sensitivity**	**HD95 (mm)**
U-Net baseline	0.79	0.72	85.2%	7.10
+ GAE	0.82	0.75	86.7%	6.12
+ GAE + attention	0.84	0.77	87.9%	5.48
+ GAE + attention + k-means ROI	0.85	0.78	88.3%	4.89
Full GAME-Net ensemble	0.91	0.84	90.1%	4.52

These results confirm that each architectural component contributes measurably to overall performance. The generative autoencoder enhances feature separability, attention mechanisms improve boundary precision, latent k-means ROI generation reduces false positive regions, and classifier gating increases robustness by preventing superfluous segmentation. The ensemble fusion yields the highest accuracy by leveraging complementary strengths of the constituent models.

### 5.6 Discussion and limitations

The experimental outcomes underscore the effectiveness of combining Generative Autoencoders, attention mechanisms, and ensemble learning for brain tumor segmentation. The robust improvements in segmentation and classification metrics demonstrate the advantage of incorporating unsupervised feature learning within a unified deep learning pipeline. Comparative analysis with recent literature confirms the superiority of the proposed approach, particularly in Dice and AUC-ROC scores. However, certain limitations persist. The evaluation was conducted on a single dataset, which may affect generalizability to other imaging domains or acquisition protocols. Advanced validation procedures such as k-fold cross-validation, multi-seed re-runs, and external dataset testing were not included because the study focuses on establishing the feasibility and internal behavior of the proposed framework rather than producing an exhaustive validation analysis. The dataset used in this study follows a subject-wise structure, which reduces the risk of slice-level leakage but also imposes constraints on flexible multi-fold partitioning. The reproducible single-split evaluation aligns with common practice in preliminary BraTS-based methodological studies, yet external validation remains essential for confirming robustness across diverse imaging protocols. Future research should focus on cross-validation using multiple datasets, adaptation to diverse imaging modalities, and further exploration of explainable AI (XAI) techniques to enhance interpretability and clinical trust.

## 6 Conclusion and future work

This study introduced an advanced ensemble deep learning framework for automated brain tumor segmentation in MRI images, leveraging Generative Autoencoders, Attention Mechanisms, and Unsupervised Learning Techniques. By employing a hybrid segmentation strategy, the proposed method effectively addresses major challenges such as tumor boundary ambiguity, class imbalance, and intensity variation in MRI scans. Experimental results demonstrated significant improvements in classification and segmentation performance, with the baseline configuration achieving a Dice Coefficient of 0.85, Jaccard Index of 0.78, classification accuracy of 87.18%, sensitivity of 88.3%, specificity of 86.5%, and an AUC-ROC of 0.91. The full ensemble variant (attention + ROI integration) further increases Dice to 0.91 on the same split (see Section 5). The proposed methodology also integrated unsupervised pre-training and early stopping to curb unnecessary computational overhead, although a detailed investigation of computational efficiency remains for future work. Training convergence analysis affirmed the stability and generalizability of the framework across diverse MRI sequences. Overall, the findings highlight the potential of AI-driven automated segmentation to support radiologists, reduce manual workload, and enhance diagnostic accuracy for brain tumor detection in clinical settings.

### 6.1 Future work

Future efforts will prioritize computational efficiency for real-time use through lightweight backbones, pruning, and quantization; investigate Vision Transformers (ViTs) and attention-based designs to enhance feature extraction and tumor boundary precision; and extend evaluation to multi-modal MRI (T1, T2, FLAIR) with cross-dataset, large-scale external validation to ensure generalizability. Integration of explainable AI (e.g., Grad-CAM, SHAP) will be pursued to improve clinical interpretability and facilitate deployment within routine workflows. These directions align with recent advances in medical AI across imaging and biosignal domains, emphasizing the importance of robust architectures, rigorous validation, and translational readiness (Anas et al., 2024; Ahmad et al., 2023; Haq et al., 2023; Alkahtani et al., 2024; Haq et al., 2025).

## Data Availability

The original contributions presented in the study are included in the article/supplementary material, further inquiries can be directed to the corresponding author.
